# Book Reviews

**Published:** 1913-11

**Authors:** 


### BOOK REVIEWS

### A PRACTICAL TREATISE ON MEDICAL DIAGNOSIS.

—For Students and physicians. By John H. Musser, M.D., LL.D., late Professor of Clinical Medicine in the University of Pennsylvania; formerly President of the American Medical Association, etc. New (sixth) edition, revised by John H. Musser, Jr., B.S., M.D., Instructor in Medicine in the University of Pennsylvania; Assistant Physician to the Philadelphia Hospital; Physician to the Medical Dispensary of the Presbyterian

DIET LISTS OF THE PRESBYTERIAN HOSPITAL, NEW YORK CITY.—Compiled, with notes, by Herbert S. Carter, M.D., Assistant Visiting Physician to the Presbyterian Hospital, Associate in Medicine at Columbia University, etc. 12mo. of 129 pages. Philadelphia and London: W. B. Saunders Company, 1913. Cloth, $1.00 net.

The diet lists contained in this volume have been prepared primarily for use in the Presbyterian Hospital; subsequently the comments on the different diets were added. The explanatory notes add to the completeness of the presentation and will be found useful.

